# A diagnostic illusory? The case of distinguishing between “vegetative” and “minimally conscious” states

**DOI:** 10.1016/j.socscimed.2014.06.036

**Published:** 2014-09

**Authors:** Sarah Nettleton, Jenny Kitzinger, Celia Kitzinger

**Affiliations:** a Department of Sociology, University of York, Heslington, York, YO10 5DD, UK; b School of Journalism, Media and Cultural Studies, Cardiff University, Bute Building, King Edward VII Avenue, Cardiff, CF10 3NB, UK

**Keywords:** Ambivalence, Diagnosis, Qualitative, Minimally conscious state, Vegetative

## Abstract

Throughout affluent societies there are growing numbers of people who survive severe brain injuries only to be left with long-term chronic disorders of consciousness. This patient group who exist betwixt and between life and death are variously diagnosed as in ‘comatose’, ‘vegetative’, and, more recently, ‘minimally conscious’ states. Drawing on a nascent body of sociological work in this field and developments in the sociology of diagnosis in concert with Bauman's thesis of ‘ambivalence’ and Turner's work on ‘liminality’, this article proposes a concept we label as *diagnostic illusory* in order to capture the ambiguities, nuanced complexities and tensions that the biomedical imperative to name and classify these patients give rise to. Our concept emerged through a reading of debates within medical journals alongside an analysis of qualitative data generated by way of a study of accounts of those close to patients: primarily relatives (*N* = 51); neurologists (*N* = 4); lawyers (*N* = 2); and others (*N* = 5) involved in their health care in the UK.

## Highlights

• Offers a novel sociological concept of ‘diagnostic illusory.’
• Use of original qualitative research on chronic disorders of consciousness to buttress this concept.
• Highlights the diagnostic operations and consequent ambivalence and tension faced by carers and practitioners.
• Extends debates in the sociology of diagnosis.

## 1 Introduction

This article seeks to propose a novel concept – *diagnostic illusory* – in order to capture the ambiguities and nuanced complexities associated with the biomedical imperative to name and classify. We suggest that diagnosis is something of a modernist notion, rooted in the idea that we can have bounded, stable and more precise diagnostic categories identified by increasingly sophisticated technologies. In more and more areas of medicine – for example, breast cancer (Curtis et al., 2012) and dementia (Richards and Brayne, 2010) – we are witnessing the sub-categorization of diagnoses, and although sociologists have demonstrated the unstable nature of diagnostic categories (Mol, 2002; Buscher et al., 2010), the lure of technological innovations in, for instance, genetics and neuroscience, that offer the promise of greater diagnostic precision remains strong (Borup et al., 2006). However, such diagnostic fine-tuning may, rather ironically, harbour unintended consequences; the imperative for diagnostic conviction could generate as many anomalies as it seeks to resolve. Moreover, diagnostic certainty could, in some instances, exacerbate existential doubt.

To ground this theorization we draw on empirical research into chronic disorders of consciousness (CDoCs) and in particular the circumstances of patients who have survived severe brain injury yet remain in long-term vegetative or minimally conscious states. The study provides a window through which we might understand this contemporary trend within medicine. It was through the analysis of our data, informed by insights from the sociology of diagnosis, that our concept took shape. We begin by introducing the extant literature on the sociology of (what are loosely and controversially called) ‘vegetative’ states and the sociology of diagnosis. We then introduce diagnostic categories applied in the context of CDoCs focusing on the distinction between vegetative states (VS) and minimally conscious states (MCS), outline their prognostic, legal, and social consequences and explore debates within the associated biomedical literatures. Turning to our empirical material we reveal conundrums associated with the determination of consciousness found amongst the views of relatives, carers and clinical practitioners. Drawing on concepts of ambivalence (Bauman, 1991) and liminality (Turner, 1967) we conclude with a discussion on the ways in which a biomedical ontology of ‘consciousness’ reifies its existence as a ‘thing’ (Taussig, 1980) that can be detected and ‘seen’ within the brain, and how this in turn generates ambiguities for those who care for and care about these patients.

### 1.1 Sociology of chronic disorders of consciousness

The survival of patients who have sustained severe brain injuries and who are (at least initially) unable to breathe or swallow is a recent phenomenon. Forty years ago they would have died relatively quickly. In many affluent societies, as the result of technologies such as, mechanical ventilators and improvements in the clinical delivery of nutrition and hydration, in concert with a medico-legal imperative to preserve life, a growing number of patients with CDoC survive for years and sometimes decades. Articles report on the reactions of care givers and the socio-legal and ethical implications of their views (Kuehlmeyer et al., 2012; Samuel and Kitzinger, 2013; Kitzinger and Kitzinger, 2013; Halliday et al., 2014), however as yet, there is only a small literature on their conceptual significance (Ben-David and Israeli, 2010; Kaufman, 2003, 2005) and it is with these theoretical contributions that we seek to engage. From these, and related studies on ‘brain death’ (Giacomini, 1997; Lock, 2002; Kaufman and Morgan, 2005), it is evident that professionals, families and wider publics struggle to make sense of patients who are neither fully ‘alive’ nor unambiguously ‘dead’. Kaufman's (2005) ethnography of North American hospital units where health workers and relatives care for patients in ‘vegetative states’ is instructive. She demonstrates how this growing patient population trouble ontologies of life and death, and challenge Western notions of personhood. New categories of patients in the uncharted territory betwixt and between life and death exist in what Kaufman calls the ‘gray zone’, that is ‘… states of being that are neither “comatose” nor “awake” or “alert,” taken together, have created zones of indistinction’ (Kaufman, 2005: 62).

Timmermans' (2005) concept of ‘death brokering’ is also useful here, since it captures the ways in which medical experts work to make these, and other ever more diversified modes of allowing or delaying death, meaningful. Health professionals:

‘offer increasingly flexible cultural scripts to render the end-of-life socially meaningful while accentuating death's existential ambiguity. Medical professionals help to create the ambiguity they promise to resolve, reinforcing the cultural need for more expert death brokering’ (p. 993).

Attempts to further demarcate and categorize ‘anomalous’ patients within the ‘grey zone’ provide one such example of medical efforts to ‘resolve’ ambiguities. Indeed, since Kaufman carried out her fieldwork in the 1990s, a new label has been applied to those who are neither ‘vegetative’ nor fully ‘conscious’ but ‘minimally conscious’ (Giacino et al., 2002). In this liminal landscape diagnostic categories are currently in the making (see RCP, 2013). Thus turning the sociological lens on to these processes is timely.

### 1.2 Sociology of diagnosis

The subfield ‘sociology of diagnosis’ (Jutel, 2011; McGann and Hutson, 2011) urges us to ‘see diagnosis as a kind of focal point where numerous interests, anxieties, values, knowledge, practices and other factors merge and converge’ (Jutel and Nettleton, 2011: 798). Diagnosis is at once a *category* and a *process* (Blaxter, 1978) that carries social, moral, economic, political as well as prognostic *consequences*. Diagnosis is a noun, a label that can serve as an apparently stable descriptor of a discrete condition. But diagnosis is also a verb that implies the act of diagnosing and is deeply embedded in our notions of medical work. As Rosenberg (2002) argues, diagnosis throughout the 20th century came to be understood as objective descriptor of a disease that, in turn, had a correspondent pathological lesion. Diseases and diagnoses he writes became ‘entities existing outside the unique manifestations of illness in particular men and women’ (p. 237). Once encoded in classificatory systems such as the International Classification of Diseases (ICD), a diagnosis feeds back into the diagnostic process (Hacking, 1999). The process is dialectical; clinically accepted diagnostic categories found in medical texts and diagnostic manuals inform day to day diagnostic work within the clinic and vice versa. The diagnostic categories of, and diagnostic ‘work’ associated with, VS and MCS should therefore be understood as an amalgam of practices that circulate throughout medical texts, scientists, clinical practitioners, relatives, bureaucrats, and patients (cf Foucault, 1980; Buscher et al. 2010; Mesman, 2008). As such it is a worthwhile line of inquiry and one we follow in our analysis of the contemporary concretization of VS and MCS. We explore what Bowker and Star (2000: 44) refer to as the ‘practical politics of classifying.’

‘Someone, somewhere, must decide and argue over the minutiae of classifying and standardizing. The negotiations themselves form the basis for a fascinating practical ontology – our favorite example is when is someone really alive? Is it breathing, attempts at breathing, or movement? How long must each of those last? Whose voice will determine the outcome is sometimes an exercise of pure power’.

The implication here is that determining evidence of ‘life’ and ‘death’ is (at the risk of understatement) difficult. Our attention is on the relatively new landscape of ‘death in life’ (Kaufman, 2005: 7) where the determination of consciousness within these borderlands has come to carry significant prognostic, legal, ethical and social consequences.

### 1.3 MCS and VS: categories and consequences

In the UK, a VS is formally defined in guidelines (RCP, 2013) as ‘permanent’ [PVS] a year after traumatic and six months after non-traumatic brain injury (in the USA the equivalent time after non-traumatic injury is three months). The diagnostic label ‘minimally conscious state’ (MCS) was ‘invented’ in 2002 by neurologists in the USA who sought to label a subgroup patients, who did not ‘fit’ the criteria of the VS, precisely because they appeared to manifest awareness, albeit at a low level and intermittently (Giacino et al., 2002). Formally described as ‘minimally responsive states’, the semantic shift from ‘responsiveness’ to ‘consciousness’ is significant because of the socio-cultural resonances and because it serves to contribute to the reification of ‘consciousness’ as a ‘thing’ (Taussig, 1980).

Attempting to assess whether the patient is in a VS or MCS is relatively routine in practice across ‘the West.’ There are calls to subdivide the MCS diagnostic category still further. Bruno et al. (2011) propose sub-categorization into minimally conscious PLUS (MCS+) and minimally conscious MINUS (MCS−) to reflect degrees of complexity of observed behavioral responses. MCS− is defined as closer to the ‘vegetative’ state – a state also referred to as ‘unresponsive wakefulness syndrome’ (UWS) (Laureys et al., 2010) to avoid the negative connotations of ‘vegetative’, and allow for the possibility that unresponsive patients *may* have some level of awareness albeit inaccessible during clinical observations.

Current clinical assessment in the UK predominantly relies on two tools: the Wessex Head Injury Matrix (WHIM) and the Sensory Modality Assessment and Rehabilitation Technique (SMART). Diagnosis based on the latter is now required in English court cases for treatment withdrawal. SMART is a formal assessment conducted in ten sessions over a three week period and is designed to provide the opportunity for patients to exhibit their full behavioral repertoire in each sensory modality. A range of standardized auditory stimuli is presented, including loud sound, voice and verbal instructions. Patients are scored at one of five levels for each modality, from “no response” or “reflex response” at the lower end of awareness to “differentiating response” at level five at which a patient may follow instructions or use an object (e.g. a pen) appropriately. The ability of these assessment tools to determine awareness is contested with claims that there are high levels of misdiagnosis (Andrews et al., 1996; Gill-Thwaites, 2004). Studies assess their relative ‘accuracy’; for example Schnakers et al. (2009) report that the Coma Recovery Scale-Revised (CRS-R) found the proportion of patients diagnosed with MCS by the CRS-R was significantly higher compared to other neurobehavioral scales such as, the ‘Glasgow Coma Scale’, the ‘Full Outline of UnResponsiveness’ and the ‘WHIM’. The relative merits of these scales are outwith the scope of this paper. What is important to note however, is that these clinical tools take behavioral responsiveness to be a proxy for consciousness. By contrast, recent diagnostic techniques that use imaging technologies presume that evidence of consciousness is to be found within the brain.

There is a growing body of work in neuroscience that advances imaging technologies as a means of providing tangible evidence of consciousness, which have the potential to overcome the vagaries of behavioral assessments (Brukamp, 2013). Monti et al. (2010) point to the way that current assessments based on clinical histories rely on ‘subjective observation’ of ‘patient's spontaneous and elicited behaviour’.

‘Differentiating between awareness and non-awareness ultimately relies on a pragmatic principle that someone is conscious if they can indicate so’ (Monti et al., 2010: 294).

This difficulty, it is argued, can be compounded by patients' sensory or auditory impairments that might mask awareness, and by the ‘intermittent’ nature of the consciousness of some brain injured patients (Monti et al., 2010: 293). While the merits and demerits of the technologies are hotly debated, there is nevertheless a strong thread that points to the brain as a locus of consciousness and a drive towards the view that functional neuroimaging (fMRI) and/or cerebral F-fluorodeoxyglucose (FDG) positron emission tomography (PET) has the potential to determine awareness (Owen et al., 2006; Von Wild et al., 2012; Stender et al., 2014). One team of researchers writing in *NeuroRehabilitation* posit that the ‘absence of (behavioral) proof of consciousness is not absolute proof of absence of consciousness’ (Grosseries et al., 2011: 5); moreover neuroimaging studies have ‘demonstrated that a small subset of unresponsive “vegetative” patients may show *unambiguous signs of consciousness* and command following [which are] inaccessible to bedside clinical examination’ (Grosseries et al., 2011: 9 *italics our emphasis*). To be sure, developments in this field are evolving rapidly with claims and counterclaims on the feasibility and use of these technologies (Coleman et al., 2009; Jox and Kuehlmeyer, 2013; Turner-Stokes et al., 2013). Nevertheless, a sociological reading might lead us to question whether this aspiration to diagnostic ‘accuracy’ runs the risk of bracketing out the nuanced issues that surround understandings of consciousness and so reify ‘it’ as a ‘thing’ that can be ‘seen’ through increasingly high-tech ‘resolutions’ (Cohn, 2004). This is salient in a wider context wherein there is a growing media interest in, and popular appetite for, neuroscientific explanations of disease, health, and indeed life (Racine et al., 2010). It is also important because diagnostic certitude can be consequential: prognostically, legally, ethically, and socially.

Placing patients on either side of the VS/MCS binary has implications for anticipated outcomes, treatments and care. MCS patients are eligible for rehabilitation therapies and pain medication regimens that may not be considered appropriate for those classified as VS. Legally, patients diagnosed as PVS, if approved by the courts, may be allowed to die through the withdrawal of artificial nutrition and hydration (ANH). By contrast (in the UK), this has never been approved for patients diagnosed as MCS (Huxtable, 2013). In England, there has been only one case of a patient diagnosed as MCS brought to court for authorization of withdrawal of ANH: the Court of Protection ruled that withdrawal would be unlawful (*W v. M* EWHC 2443 (Fam). 2011). It was judged that ‘the importance of preserving life is the decisive factor in this case’ (ibid – paragraph 249). Despite so much resting on these diagnostic categorizations closer analysis of the views of relatives and clinicians reveals that diagnostic clarity in practice is far from straightforward and fails to resolve ambiguities and does not address their quest for meaning. We expand on these issues below following a description of our study and methods.

## 2 Study and methods

The empirical research reported here is part of an ongoing interdisciplinary project that comprises reviews of documentary sources such as: biomedical literatures, legal judgments, and media reports, and qualitative interviews undertaken with 51 relatives of patients who are (or were) in a CDoC, four neurologists, two lawyers and five other relevant professionals (e.g. a care home manager and a physiotherapist). Research ethics committees at the Universities of York and Cardiff approved the study, as did the NHS (NHS REC reference number: 12/SC/0495).

Relatives were recruited through advertising via brain-injury support groups, care homes and websites and through contacts following formal presentations about the research. They vary in terms of age, gender, ethnicity, and socio-economic status, as do the patients. All participants were interviewed by either the second or the third author, and were mostly one-to-one, but occasionally in pairs. Interviewees were mostly parents, siblings, spouses⁄partners and adult children of the patient. Most patients were currently formally identified as either VS or MCS (some had died by the time of interview; others had emerged with severe neurological deficits). The topic guide was flexible to allow people to tell their stories. Interviews usually lasted between two and four hours. Pseudonyms are used throughout. Interview transcriptions were discussed by the research team, coded to facilitate retrieval and to help identify themes. Issues pertaining to diagnosis were coded e.g. disclosure of diagnosis, diagnostic terms, references to diagnostic criteria (e.g. eyes, interpretation of bodily movements and so on).

Our analytic strategy was also to ‘use the data think with’ (Hammersley and Atkinson, 2007: 163) and to reflect sociologically on the assumptions and interpretations that circulate in relation to the newly emerging categorizations of VS and MCSs. Our analytic orientation is informed by Bird-David and Israeli's (2010) relational approach and presumes that patients as persons may be constructed through interactions with those around them. Moreover, we work on the presumption that patients who have limited or no consciousness are agential in the sense that they invoke responses in people and things that surround them. It is to these responses and search for meanings to which we now turn.

## 3 Determining consciousness: but what does it mean?

As we have seen within the neuroscience literature there are researchers who point to the limitations of clinical assessment tools for the diagnosis of VS and MCS, and who advance the potential of imaging technologies (Sleigh and Warnaby, 2014). Such debates are not unique to the study of CDoCs and these technologies have been studied by sociologists in other medical settings, who document how the images are open to multiple interpretations even amongst those working within the same medical specialty (Dumit, 1999; Joyce, 2008). Such issues are pertinent here, but in our particular case there are further complications. The fact that fMRIs are rarely practicable for many VS and MCS patients (because their bodies are mobile and the technology requires that them to be motionless) is significant, but more salient for our thesis is the metaphysical problem as to what visualizations of the brain actually mean and the lack of ontological consensus about what fMRI reveals. What is consciousness? Can it be detected in blood flow in the brain? Commenting on the scope of fMRI to determine consciousness one neurologist said in interview, ‘at the moment we have no idea of what it really means. We don't know whether it really means they're aware’. He reflects further on this.

‘How is it possible to breathe if you have nil awareness? I think philosophers and neuroscientists have not yet solved the problem of: ‘What is consciousness?’ And if they haven't solved it after hundreds of hours and papers, then I'm not going to solve it [laughs] and I have to take a rather more pragmatic view and my bottom line thinking is the question: Have they extracted meaning and/or have they demonstrated some goal-directed activity? You ask about doubt – and I suppose the only other area where I have doubt – which is more an in-principle doubt than it is a doubt on an individual patient basis – is that it is at least plausible, I presume, given that that we have low awareness states and given that we know, in low awareness states, they're often only aware for short periods of time, how do I know that this person, who has been tested as being unresponsive, doesn't have a microsecond of being aware? A microsecond. I couldn't possibly tell. What about a second? I couldn't tell that either. I'd need at least two minutes awareness to be certain that they're aware’.

Echoing this neurologist, Neil, whose son was diagnosed as VS, an official diagnosis he ‘accepts’, struggles to make sense of the metaphysics of life without consciousness.

‘What is a vegetative state? That's never been explained. We've had it up on the computer, but that's still never, ever been explained to us. You know, what a patient in a vegetative state goes through, sees, hears. I mean surely they must hear something, they must see something, or do they just not? If that's the case, then they're blind, deaf and they're just lying there aren't they? […] I mean surely his mind must recognize something. I mean I find it absolutely impossible, God in heaven, that your mind doesn't recognize something. I mean – But if they're saying he doesn't, then he doesn't’.

Thus Neil at the same time as he accepts the validity of the VS diagnosis finds it ontologically perplexing (‘absolutely impossible’). This ambiguity is evident in other areas of medicine where medical diagnoses and explanations are ‘accepted’ and yet remain incomprehensible at the level of meaning. Comaroff and Maguire (1981) found that parents of children with leukemia understood the diagnosis but also found it perplexing at the level of meaning. They argue that, ‘medicine can be seen as ambiguous in a double sense: the more it appears to control, the more threatening is the domain where knowledge is still lacking; and the more it controls, the more alienated the layman himself from control over its effects’ (1981 p. 115). Indeed, as discussed above clinical scripts can generate as many questions as they resolve and so exacerbate ‘existential ambiguity’ (Timmermans, 2005). So for Neil to see his son ‘alive’ without consciousness is difficult to comprehend and he finds himself in the midst of a ‘cultural remapping of notions of “life” and “persons”’ (Kaufman 2000: 70), experiencing a disturbing mixture of what Kaufman calls ‘death within life’ which constitutes an archetypical liminality as described by Turner (1967). Liminality is a transient zone characterized by an absence of familiar norms and a destabilization of received notions of personhood. Social mores and prescriptions are suspended and routine interactions dissolve into confusion. Patients in a PVS are not experienced by their relatives as unequivocally ‘alive’ or unequivocally ‘dead’ (Holland et al., 2014) and they struggle to make sense of this. Indeed, the process requires a gestalt shift even in terms of our mundane, ingrained readings and interpretations of the human body and its movements.

### 3.1 Reading bodily movements or embodied actions

Listening to relatives' accounts it is evident that routine ways of interpreting bodily movements were bewildering. How they should ‘read’ the patient's body? How should they interpret the movements of their son, daughter, mother, partner, or spouse? How, or can, they distinguish between ‘movements’ and ‘actions’, or between ‘reflexes’ and ‘embodied reactions’? From a clinical perspective, in the days or weeks after severe brain injury, patients are diagnosed as comatose: they have their eyes closed and do not manifest sleep-wake cycles (Laureys, 2007). The move from coma to a VS is marked by eye-opening and the onset of sleep-wake cycles. In our everyday lives (and in media representations of coma) eye opening tends to be associated with ‘waking up’ and for many participants is a significant moment. Belinda, describes how her son (diagnosed as VS) would apparently respond to her in the first few months after his injury:

‘He would open his right eye, the side he was hit on. The left eye was still badly bruised and not open. It was months before that opened but his right eye would open. I would come in the room, I would go ‘Hi darling, Mum and Dad are here!’ And we'd get a response. […] He must've known I was there – or was that just a fluke, wish we knew. I don't know. […] I can't say he's completely brain dead because I don't know if he is. If he can sit in a chair with his eyes open, if he can be put to bed and shut his eyes, something's making him do that.’

Belinda (like Neil) ‘accepts’ the VS diagnosis, which by definition rules out the possibility of consciousness, yet there are hints of uncertainty. Officially her son has no awareness and yet the comment ‘he must've have known I was there’ suggests the contrary. Furthermore an apparently illusive ‘something’ making him act/move alludes to a mysterious corporeal or extracorporeal realm. Hesitation and self-questioning in such reflections in the interviews is endemic – ‘was that just a fluke’ – like Neil she does not know for sure.

Hannah gives a rather different account of the first time she saw William with his eyes open, around a month after the precipitating event. She describes it as ‘actually quite distressing’.

‘I went in, and there he was with his eyes open. And I went around – he was looking out the window, so I stood looking at him, I said, “William, it's Hannah”. And nothing. He just stared. He wasn't even focusing on anything, you know. Not like say if there was an animal there, and you look at the animal, the animal looks at you, and there's that, you know. But there was nothing’.

She recalls how a doctor had said to her that: ‘”it's like a spontaneous thing. He's not, you know, he's sort of looking but he's not seeing. We don't really think that it's of any significance as such.”’ Thus the opened eyes, the doctor explained, were not evidence of awareness, or at least ‘*we don't really think* it's of any significance.’ This is a neat illustration of the way in which, within modern health care systems, it is the medical profession who prescribe how we should read bodies. In this instance, such a reading was congruent with Hannah's and useful for the family because it reinforced a PVS diagnosis critical for the application to the courts for treatment withdrawal.

When Harry and Natalie (interviewed as a couple) talked about Harry's sister Zoe they were clear that she should be allowed to die, a position that they had come to over some years. They, like other relatives, struggle to make sense of events and although they felt sure they now know what to do for the best, this was far from straightforward. In this sense relatives resemble the ‘moral pioneers’ that Rapp (2000) has written about in the context women undergoing tests for amniocentesis. The technological affordances at the margins of birth and life give rise to circumstances wherein women ‘are forced to judge the quality of their own fetuses, making concrete and embodied decisions about the standards for entry into the human community’ (p3). Similarly, the technological affordances that place those who are alive in circumstances where the person may no longer be ‘living’ provoke comparable moral quandaries which take time to work through, as is evident in following exchange.

Natalie: So I mean that is a difficulty. That's a hurdle we've got to get over. What we are convinced about is that from everything that we can find out, it is not in Zoe's best interest to be still alive because she's existing. She isn't living.

Harry: I mean there's a line where it says clinicians are good at fixing bodies but they're not good at fixing brains. [Interviewer: Yeah. You've said she's existing, not living, and I think you said earlier that the Zoe you knew ‘died’ four years ago] Harry: She did, yeah.

Later Harry adds more poetically:

The body's like a flower. It withers and it's gone, you know. That's what's happening. A beautiful flower is withering and is going. It's just, you know, there's nothing more that can be said really. It's gone through – gone through that phase and that's it.

Conversely for relatives who want to support life and prevent death, a MCS diagnosis may bring relief. Currently this diagnosis (probably) removes the threat that the patient could be allowed to die by withdrawal of ANH and broadens the scope of rehabilitation. For these relatives the patient remains alive as is evidenced by indicators of intentionality and awareness within the anatomical frame.

Returning to the issue of eye opening, Stavros, like Hannah, recalled the day his brother first opened his eyes, but in contrast to Hannah, he imputes intentionality. Where Hannah had reported that ‘there was nothing’, Stavros comments ‘he looks at you’ and ‘there was something there’. Moreover his narrative structure implies the causal significance of his mother's visit and his brother's intentionality:

‘She sang to him, you know, cried a lot. Anyway, the very next day, Len opened his eyes wide open for the first time. Properly kept them open. Then I knew that he wanted to live. That was the instrumental thing that made me think, ‘he wants to live’, you know. And it was sort of a steady improvement. Even the doctors said that he might be a vegetable, blah, blah, blah, but you can see the way he looks at you. To me, he wasn't a person in a vegetative state. There was something there’.

In keeping with our relational analytic approach we can see the agential significance of the patient's body and helps us appreciate why diagnostic decisions are not always shared. For example, Stavros notes the doctors had at that stage classified Len as ‘potentially a vegetable’, however he is clear about his view as to Len's awareness: he ‘wanted to live’. Siobhan's account of her ‘interaction’ with her husband similarly enacted him as a knowing person.

‘He will always react when I come in and in fact yesterday I think I got a smile for the very first time in ten months. I went in, “So how are you sweetheart? How's it going?” Just as I normally do, and he turned his head as he always does and he made this facial expression. And now Bob is not a smiler (laughs) he's not a smiley person, you know. And I got this expression and I mean it's the first time I could actually say he smiled at me.’

Thus smiling, eye opening, head turning, and hand grasping can be accepted by relatives as consistent with VS but may belie other contrasting yet simultaneous interpretations. People hold multiple, shifting and what might seem, on a superficial level, contradictory views about presence of consciousness. Views are rarely static especially as relatives researched information on CDoC, discussed their views, reflected on the possible outcomes, and as the patient's body changed over time. A nuanced appreciation of the way relatives can hold inconsistent thoughts contrasts with the view of some clinicians who imply that relatives' ideas are sometimes borne of ignorance. Some claim that relatives are inclined to interpret head turns and hand grasps as intentional because they ‘misunderstand’ that such movements are congruent with an absence of consciousness (Majerus et al., 2005). Our data point to a more subtle process where relatives are working to make ontological and epistemological sense of the conundrums found within this unique liminality.

These ambivalences point to the scope for tensions that can, and inevitably will, permeate communication between relatives and clinicians. Relatives sometimes described their anger towards health professionals who they felt had acted insensitively. Some recounted conflicts that had been played out in consultations and in what are known as ‘best interest’ meetings where care plans are explored. Frustrations were also reported about the adequacy of ‘expert’ assessments carried out by neurologists who had been insufficiently thorough, and/or had failed to give sufficient consideration to the fact that it was relatives who knew what patients would have wanted. Such tensions are perhaps inevitable given the life-transformative circumstances and where a diagnosis of PVS and MCS is so important in relation to decisions about care plans, insurance, resources and the possibility of treatment withdrawal. Thus there is an imperative to determine a definitive diagnosis to facilitate decision-making and some degree of resolution, and yet at the same time diagnostic processes and outcomes are mired with ambiguity and ambivalence.

## 4 Ambiguity, ambivalance and the ‘diagnostic illusory’

Our analysis reveals just how taxing attempts to determine the presence of consciousness can be, not least because we have little in the way of shared understanding as to what consciousness ‘is’, the means of identification are contested and a diagnosis of VS or MCS is so consequential. All this takes place in the context of emotionally shattering circumstances where families experience the ‘loss’ of a loved one to a novel liminal landscape (Turner, 1967) of which most people have no prior experience. As we have seen, over the last few decades there have been attempts to bring order to this zone by classifying patients within in ever more complex grids – to label them as MCS, MCS+, MCS-, Persistent VS, Permanent VS, unresponsive wakefulness syndrome (UWS) and so on. Arguably this objective to establish ever more discrete diagnostic categories is an inherently modernist phenomenon and brings unintended consequences. It is emblematic of the ‘tyranny of diagnosis’ that Rosenberg (2002) writes about – whereby diagnostic categories have become rooted within the anatomical frame to the exclusion of the person or place. In medical specialties such as neurology and psychiatry, such technologies have meant that conditions previously identified by behavioral symptoms ‘are potentially being re-assigned as brain disorders that can be isolated in the body’ (Cohn, 2010: 66). Lock (2001) argues that the brain, more than any other anatomical organ, is becoming synonymous with ‘life’. This observation that there is a prevailing view of a correlative relation between consciousness and the brain is a ‘new thought style’ Fleck (1935) emerging from neuroscience and is permeating other discourses. Sociologists point to the ubiquity of neuroscientific theories of biological consciousness (Cohn, 2004; Buchman et al., 2013; Pickersgill, 2013) which are pervasive not only in academic circles but also in popular discourses where there is a media appetite for, and public receptivity to, them (Williams, 2009; Nuffield, 2013). An implicit credence is given to the view ‘that consciousness was merely an object lying in wait for these new technological techniques, and so disguise the very fact that they have been increasingly subjected to cultural and historical modification’ (Cohn, 2004: 58).

Thus the calls within the neuroscience literature to move beyond clinical evaluations of patients with CDoC based on ‘behaviours’ (Owen, 2008) and invest instead in ‘state-of-the-art neuroimaging methods in the assessment of patients’ (Owen and Coleman, 2008: 235) have the potential to garner support amongst relatives and wider publics. However tantalizing more precise diagnostic categorizations rooted in the depths of the brain may be, sociological studies of other related high tech innovations suggest that such aspirations to confirm an ‘aware’ person, may be illusory. As Cohn (2004) elegantly argues

‘The illusion is simple: the brain has long since been the locus of various surface knowledges that up until recently have developed relatively independently. But the functional brain image holds the promise of establishing a renewed confidence in both depth and unification towards a science of consciousness itself. Thus, the claim, described earlier, that this is the birth of a meta-science of the brain is itself embodied in the very focus upon what is seen as the essence of the corporeal person’ (2004: 60).

These technologies are indicative of a privileged biomedical ontology of consciousness only accessible to neurobiologists. A corollary may be that alternative framings, such as, relatives' views as to what patients may have wanted, or relatives' readings of bodily movements become relegated within the diagnostic process.

Bauman's (1991) notion of *ambivalence* provides further analytic purchase here, and may help us make some sociological sense of the anxieties that categorical diagnostic imperatives give rise to.

‘Ambivalence is a side product of the labor of classification; and it calls for yet more classifying effort. Though born of the naming/classifying urge, ambivalence may be fought only with a naming that is yet more exact, and classes that are yet more precisely defined: that is, with such operations as will set still together (counter-factual) demands on the discreteness and transparency of the world and thus give yet more occasion for ambiguity. The struggle against ambivalence is, therefore, both self destructive and self propelling’ (1991: 3).

In sum, the more we try to name and classify the more confused we become; the more confused we are the more we try to classify in order to manage, control, and decide what is to be done for the best. The problem is circular, and especially pertinent for a population who are alive and (possibly) conscious. Even in cases where diagnoses are made the circumstances still demand ‘cultural scripts’ and modes of ‘death brokering’ as ‘the postponement of death again carries its own existential ambiguity and anguish because it is expressed in risk factors that offer probabilities but no guaranteed results’ (Timmermans, 2005, 1007). Survivors of severe brain injury are emblematic of processes described by Timmermans:

‘Death brokering thus contains a self-fulfilling principle: medical practitioners define problems and offer solutions that never completely address the problems or raise new issues, underscoring the need for further expert involvement in the end-of-life. Medical death brokering rests on the hope that with more professional involvement dying will be improved and deaths have not been in vain’ (2005: 995).

Certainly, there is a proliferation of experts who orbit these patients such as; GPs, neurologists, nurses, scientists, lawyers, and rehabilitation specialists who engage with carers and relatives who, in turn, bring their own expertise. Each draw upon multiple sources of knowledge and empirical observations in order to better understand patients and grapple with the ‘existential ambiguity’ that the possibility, proximity and postponement of death brings (Timmermans, 2005: 993). In this respect, we can see how the creation of the MCS category in 2002 adds to these ambiguities, and is consistent with Baumen's theorization that the biomedical ‘naming/classifying urge’ in tandem with increasingly sophisticated diagnostic biotechnologies conspire (despite the best of intentions) to amplify ambivalence.

Thus the attempt to firm up diagnostic categories, to place and to name, may be a *diagnostic illusory*. The apparent stability of diagnosis not only belies its inherent instability, but a diagnosis may also fail to attend to meanings inherent in illness, life and death. As we have seen even when doctors, relatives, and lawyers agree on, or accept, a diagnosis of either VS or MCS there is a lingering omnipresence of ‘existential ambiguity’. Uncertainty and the search for meaning are unlikely to be dispelled as a result of imagings of consciousness because, as our participants ask: ‘what does it mean?”. Furthermore, sociological research indicates that diagnostic categories are rarely stable. Take Mol's (2002) ethnography of the diagnosis of atherosclerosis where she shows how the body is ‘enacted’ from varying perspectives and diagnosis is a ‘patchwork singularity’: a temporary stitching together of multiple enactments. In practice classifications are rarely tidy, consensual and fixed. Their fluidity is shaped too by decisions beyond the clinic. For example, recent court judgments in England have supported doctors' rights to withhold treatments such as cardio-pulmonary resuscitation, vasopressor drugs and renal replacement therapy from MCS patients (*Aintree University Hospitals NHS Foundation Trust v* James [2013] UKSC 67) even when the judge accepts a family's testimony that the patient himself would want them to sustain his life in MCS as long as possible and would see his own suffering as bringing him closer to God (www.theguardian.com/law/2013/nov/13/family-muslim-lose-right-to-life-case-god).

In sum, we suggest that there is an increasingly pervasive biomedical view that consciousness is a ‘thing’ (Taussig, 1980) laying latent but as yet unseen. Some neuroscientists (Grosseries et al., 2011; Stender et al., 2014) suggest that consciousness may be present in patients labeled as vegetative and so trouble the diagnostic boundaries of VS. Our argument is not that diagnosis is inherently and invariably a bad thing: a label can be useful for accessing resources and/or assessing outcomes, We posit however, that there is a need to give consideration to the wider ramifications and inherent tensions associated with diagnostic categories and processes which may, in turn help us appreciate some of their unintended and unanticipated consequences. For example, diagnosis may ultimately fail to quell doubt. There may be other implications too. The diagnostic net could be widened with increasing numbers of patients being kept alive, ‘just in case’ they ‘are’ conscious, regardless of what relatives, or the patients themselves might have wanted or resources allow. These ‘high-tech’ means that seek to resolve ambiguities harbor the potential to introduce new complexities (Samuel and Kitzinger, 2013), mobilize discourses of ‘hope’ and create a mirage of diagnostic certainty which, in turn, could also facilitate a privileging of biomedical readings of the body over competing lay, clinical or other interpretations.

## 5 Conclusion

Although we have unparalleled knowledge and means to preserve, modify and regulate human bodies, we live in an era of unprecedented doubt as to what bodies and minds are, how we might understand them, and how they relate to our notions of personhood (Shilling, 2012). We live too, in an era characterized by perpetual liminality and ambivalence, as certainties (such as death) are configured as choices: where, when, and on whose authority can we decide to permit death. Technological innovation, in tandem with medico-legal imperatives to preserve life, have resulted in longer survival rates, and patients variously diagnosed as ‘comatose’, or in ‘unresponsive wakefulness syndrome’, ‘persistent vegetative’, ‘permanent vegetative’, and ‘minimally conscious’ states with each label carrying varying possibilities. From an analysis of medical literatures, in tandem with interview data interpreted with the help of Bauman's thesis on ambivalence and Turner's work on liminality, we tentatively propose the notion of a *diagnostic illusory*: the idea that this modernist urge to classify and name may serve to exacerbate (or at the very least fail to address) the existential challenges faced by relatives and carers and, moreover, raise hopes and expectations of clarity which may be unfulfilled. While a definitive diagnosis may be useful for relatives and health care staff to improve prognostic accuracy and secure resources (such as rehabilitation for MCS patients) or particular outcomes (such as withdrawal of ANH for PVS patients) it seems unlikely that it could address the ‘ambiguity and search for meaning’ (Comaroff and Maguire, 1981: 115). Although we have generated the concept of *diagnostic illusory* from a case study of CDoCs we hypothesis that the concept may be generalizable to other conditions and settings not least because disease and diagnostic boundaries are ever more fluid and permeable with the convergence of risk and disease (Aronowitz, 2009). Moreover, from the lessons learned here we would caution against an elision of the biomedical gaze with a neuro-technological imperative that through the recourse to popular discourses on neurobiology, offer hope and increased expectations (Borup et al., 2006) that consciousness exists within the depths of the brain and we will in the future be able to unambiguously identify it. Whilst there is no doubt that a diagnosis of MCS or VS can bring relief for relatives and possibly (but who can know) for patients it is unlikely to fully ever extinguish ambivalence and doubt. In this regard diagnoses can comprise a mirage of certainty we call *diagnostic illusory*.
